# Optimization of cultural conditions for conversion of glycerol to ethanol by *Enterobacter aerogenes* S012

**DOI:** 10.1186/2191-0855-3-12

**Published:** 2013-02-06

**Authors:** Raymond E S Nwachukwu, Abolghasem Shahbazi, Lijun Wang, Mulumebet Worku, Salam Ibrahim, Keith Schimmel

**Affiliations:** 1 Energy and Environmental Systems, Sockwell Hall North Carolina A&T State University, 1601 East Market Street, Greensboro, NC, 27411, USA; 2 Biological Engineering, North Carolina Asss&T State University, Greensboro, NC, USA; 3 Department of Animal Sciences, North Carolina A&T State University, Greensboro, NC, USA; 4 Food and Nutritional Sciences, North Carolina A&T State University, Greensboro, NC, USA

**Keywords:** *Enterobacter aerogenes*, Ethanol, Glycerol, Metabolic pathway, Fermentation

## Abstract

The aim of this research is to optimize the cultural conditions for the conversion of glycerol to ethanol by *Enterobacter aerogenes* S012. Taguchi method was used to screen the cultural conditions based on their signal to noise ratio (SN). Temperature (°C), agitation speed (rpm) and time (h) were found to have the highest influence on both glycerol utilization and ethanol production by the organism while pH had the lowest. Full factorial design, statistical analysis, and regression model equation were used to optimize the selected cultural parameters for maximum ethanol production. The result showed that fermentation at 38°C and 200 rpm for 48 h would be ideal for the bacteria to produce maximum amount of ethanol from glycerol. At these optimum conditions, ethanol production, yield and productivity were 25.4 g/l, 0.53 g/l/h, and 1.12 mol/mol-glycerol, repectively. Ethanol production increased to 26.5 g/l while yield and productivity decreased to 1.04 mol/mol-glycerol and 0.37 g/l/h, respectively, after 72 h. Analysis of the fermentation products was performed using HPLC, while anaerobic condition was created by purging the fermentation vessel with nitrogen gas.

## Introduction

Fossil fuels are the major sources of energy, and they account for about 80% of global energy demand (Sarma et al. 2012). But they are characterized with a lot of problems, which include non-renewability, erratic prices, global warming, ecosystem imbalance, health hazards, and other environmental/agricultural effects like pollution and food shortage. Therefore, there is need for a renewable, healthier, more environment friendly, abundant/secure, and sustainable alternatives. Biofuels potentially provide these advantages and are increasing in global demand (Sarma et al. 2012).

Biodiesel is produced by alkali-catalyzed methanolysis or ethanolysis of triglycerides, a process known as transesterification. During esterification, glycerol, which amounts to 10% of produced biodiesel, is generated as byproduct. Biodiesel production is increasing annually. The top five global producers of biodiesel are Argentina, Brazil, France, Germany, and the United States of America (Sarma et al. 2012). In the United States, which is second only to Germany in global production (Sims 2011), biodiesel production increased from 1.9 million liters in 1999 to 568 million liters in 2006 (Pachauri & He 2006). By 2009, 15 billion liters were produced (Ghosh et al. 2012). It is estimated that the global biodiesel market will reach 140 billion liters by 2016, with an annual growth of 42% (Fan et al. 2010), and that the global annual production would be about 159 billion liters by 2020 (OECD/FAO 2011). This means that about 16 billion liters of global crude glycerol will be generated annually by 2020, since crude glycerol surplus is proportional to biodiesel production.

Glycerol is a trihydric alcohol, namely 1,2,3-propanetriol, and a natural constituent of animal and plant lipids. It finds wide applications in industries such as food and drinks, toothpaste, cosmetics, toiletries, plastics, tobacco, pulp and paper, paint, leather and textile, pharmaceuticals, and automotive (Choi 2008; Nicol et al. 2012; Rossi et al. 2012). Recently, it is being studied as potential substitute for carbohydrate sugars in industrial fermentation processes to obtain a broad range of value-added organic products (Rossi et al. 2012). It is also released from the triglycerides by processes other than transesterification, such as high-pressure splitting and saponification (Hazimah et al. 2003; Nicol et al. 2012): it is a byproduct of bioethanol production process (Choi et al. 2011), and it is a byproduct of alcoholic beverage production (winery and brewery) from sugars by yeasts (Chapman et al. 1995).

The surplus of crude glycerol has led to over 10-fold fall in the price of industrial glycerol in the last few years (Ghosh et al. 2012), resulting in the shutdown of some glycerol production plants like Dow Chemicals (McCoy 2006). Apart from the fall in economic/market value of industrial glycerol, crude glycerol surplus presents other challenges. First, it cannot be applied in any industrial process without purification. The impurities are as diverse as the feedstock and catalysts used in the transesterification process, and may require complex and expensive processes to remove. Second, crude glycerol cannot be disposed into the environment without treatment, and the cost of such treatment could be prohibitive. Methanol, one of the impurities in crude glycerol, is known to be a toxic alcohol (Selembo et al. 2009). It can affect soil microbial flora if crude glycerol is disposed in the environment. It can contaminate ground waters. The sodium or potassium hydroxide residue of the crude glycerol gives it elevated pH, which may endanger biotic community if crude glycerol is disposed without neutralization. Besides, action of indigenous microorganisms will release offensive odor that pollutes the atmospheric environment (Sarma et al. 2012). Therefore, crude glycerol must be treated before disposal, an economically inefficient and ineffective process for biodiesel industries.

To improve the economic viability of biodiesel production, therefore, development of environmentally responsible processes to convert the low-priced glycerol byproduct to higher value products is expedient. Moreover, the twin facts of its increasing abundance and attractive low pricing make crude glycerol a potential sustainable feedstock for producing value-added commercial compounds (Fan et al. 2010).

Many chemical and biological processes are being explored to derive economic and environmental benefits from crude glycerol (Nicol et al. 2012), but biological means is safer, cheaper and more environment friendly. Thus, Choi et al. ( 2011) defined glycerol-based refinaries as the microbial fermentation processes that use surplus, inexpensive crude glycerol as feedstock to produce fuels and chemicals. The biological agent may be algae, fungi, or bacteria. *Enterobacter aerogenes*, a member of *Enterobacteriaceae*, has been shown to produce predominantly ethanol and hydrogen from glycerol (Ito et al. 2005). Nwachukwu et al. ( 2012) reported that *E. aerogenes* S012, a mutant of *E. aerogenes* ATCC 13048, produced over 20 g/l ethanol from glycerol.

Ethanol is primarily produced from corn in the United States. This is an unsustainable practice because corn is a food crop. There is, therefore, need for alternative sources of feedstock for bioethanol production. Glycerol promises to be one of such feedstock. In comparison of the processes, bioethanol production from crude glycerol could be more economical than the use of corn or lignocellulosic biomass (Choi 2008). Also, the cost of ethanol production from glycerol is about 40% less than when produced from corn (Yazdani and Gonzalez 2007). First, crude glycerol is a cheaper feedstock than corn; second, building and operating anaerobic fermentors is cheaper in cost and energy (Yazdani and Gonzalez 2007). It is predicted that glycerol-based ethanol may enter the market earlier than cellulosic ethanol because the latter is still believed to be some years away (Choi 2008).

The primary aim of this paper is to identify, through design of experiments, the cultural factors that have greatest effect on the production of ethanol from glycerol by *E. aerogenes* S012. The paper also seeks to develop a statistically sound regression model that establishes the relationship between the factors (process variables) and the ethanol output (response variable). Finally, information from the model equation will be used to optimize those process factors for high production of ethanol from glycerol.

## Materials and method

### The inoculum

*Enterobacter aerogenes* S012 – a mutant of *E. aerogenes* ATCC 13048 (Nwachukwu et al., 2012) – was cultivated using sterilized regular trypticase soy broth (n-TSB) and agar (n-TSA) (BD Difco Laboratories, Sparks, Maryland, USA). Sterility was achieved by autoclaving the media for 15 min at 121°C. The organism was first grown for 24 h at 37°C in n-TSB from where it was cultivated on n-TSA plates by streaking, to establish purity. A pure colony on the plate was subcultured on fresh sterile n-TSB for 24 h at 37°C, kept at 4°C, and used to prepare inocula for the fermentations. The inoculum was prepared by aseptically inoculating an 18–24 h culture of the organism into a sterile 100 ml Tryptic Soy Broth without Dextrose (TSB) (BD Difco Laboratories, Sparks, Maryland, USA) containing 20 g/l glycerol in a 250 ml Erlenmeyer flask. This was again incubated for 18–24 h at 37°C, then washed thrice with 0.1% peptone water and re-suspended in sterile TSB with the same glycerol concentration as the fermentation medium. It was then used to aseptically inoculate the fermentation broth such that the inoculum made up 5% of the broth.

### Analytical methods

Samples were taken and analyzed for succinic acid, lactic acid, acetic acid, ethanol, 1,3-propanediol, and glycerol by liquid chromatography. A High Performance Liquid Chromatograph, HPLC (Waters Corporation, Milford, USA), equipped with a Shodex RSpak SH-1821 column (temperature, 70°C) and refractive index (RI) detector (temperature, 40°C) was used. The mobile phase was 0.01 N H_2_SO_4_ with flow rate of 1.0 ml/min.

### Design of experiments

Two experimental design methods were used for the screening and optimization of process variables.

### Taguchi method

Taguchi experimental design method (Fraley et al. 2007) was used to screen four process variables—temperature (T), pH (P), agitation speed (A), and inoculum volume (I)—each at three levels. From the orthogonal array selector, L9 Array was selected since the highest level for the factors is 3. Therefore, triplicates of nine experiments, as described in Table 1, were performed and the results used to determine the signal-to-noise (SN) ratio and range (R) for ethanol production after 24 h and 120 h. The values of R were ranked in increasing order, 1 being the highest and 4 the lowest. A factor with high range of SN means it has high signals and low noise; a factor with a low range of SN means low signal and high noise. The factors ranked 1–2 were selected for optimization since they are the ones with the greatest effect on the response variable, namely ethanol production.

**Table 1:** Taguchi L9 orthogonal array

**Experiment**	**T**	**A**	**P**	**I**
1	1	1	1	1
2	1	2	2	2
3	1	3	3	3
4	2	1	2	3
5	2	2	3	1
6	2	3	1	2
7	3	1	3	2
8	3	2	1	3
9	3	3	2	1

^a^Abbreviations: *Gly* – glycerol, *Suc* – succinate, *Lac* – lactate, *Ace* – acetate, *1,3-Pdo* – 1,3-propanediol, *EtOH* – ethanol, *Ae* – aerobic, *An* – anaerobic, *1dAn* – 1 day anaerobic process.

^b^T1, T2, T3, = 30°C, 35°C, and 40°C; A1, A2, and A3 = 120 rpm, 160 rpm, 200 rpm; P1, P2, and P3 = 6.0, 6.5, 7.0; and I1, I2, I3 = 3%, 4%, 5%.

The following formula was used to calculate SN where y_i_-bar is the mean value, s_i_ the variance, and y_i_ the value of the performance characteristic for each given experiment.

$$\mathrm{SNi}=10\log\frac{{\bar{y_{i}}}^{2}}{{s_{i}}^{2}}$$

Where

$$\begin{array}{l} \bar{y_{i}}=\frac{1}{N_{i}}\sum_{u=1}^{N_{i}}y_{i,u}\\ s_{i}^{2}=\frac{1}{N_{i}\;-\;1}\sum_{u=1}^{N_{i}}\left(y_{i,u}-\bar{y_{i}}\right) \end{array}$$

i=Experiment number

u=Trial number

N_i_=Number of trials for experiment i

### Full factorial design

A full factorial experimental design was employed to investigate the effect, and choose the optimum values, of temperature (T), agitation speed (A), and time (M), on the conversion of glycerol to ethanol. Each factor was tested at three levels in duplicates. Using the formula k^n^, where k = level and n = number of factors, 3^3^ experiments were performed in duplicates, and the average values recorded. This gives a total of 54 experiments. The levels of temperature were 30°C, 35°C, and 40°C; levels of time were 24 h, 48 h and 72 h; and levels of agitation speed were 120 rpm, 160 rpm and 200 rpm. The full factorial design matrix showing the variables is presented in Table 2. Linear regression and analysis of variance (ANOVA) were conducted to identify the process variables that have significant effect on the response (i.e., *p* < 0.05), and establish regressional relationship between the process variables and the response variable. All statistical analyses were performed using Microsoft Excel 2007 software. The following second-order polynomial function was used to predict the optimum conditions:

(1) $$Y=\beta_{0+}\sum \beta_{i}X_{i}+\sum \beta_{\mathrm{ii}}X^{2}+\sum \beta_{\mathrm{ij}}X_{i}X_{j}$$

where Y is the predicted response; X_i_ and X_j_, the independent variables; β_0_, β_i_, β_ii_, and β_ij_, the intercept, linear, quadratic, and cross-product (interaction) coefficients, respectively.

**Table 2:** Effect of Temperature, Time, and Agitation on ethanol production from glycerol

**Run**	**Time (h)**	**Temperature (°C)**	**Agitation (rpm)**	**Ethanol (g/l)**
1	24	30	120	7.48
2	48	30	120	11.72
3	72	30	120	15.53
4	24	35	120	11.51
5	48	35	120	17.02
6	72	35	120	18.83
7	24	40	120	12.56
8	48	40	120	16.96
9	72	40	120	17.97
10	24	30	160	4.15
11	48	30	160	9.11
12	72	30	160	12.88
13	24	35	160	8.01
14	48	35	160	12.9
15	72	35	160	15.34
16	24	40	160	10.24
17	48	40	160	13.31
18	72	40	160	13.88
19	24	30	200	9.52
20	48	30	200	14.92
21	72	30	200	16.65
22	24	35	200	12.76
23	48	35	200	16.97
24	72	35	200	17.19
25	24	40	200	13.71
26	48	40	200	16.86
27	72	40	200	17.32

### Fermentation broth

The fermentation medium for the screening of process variables was prepared by mixing 30 g/l pure glycerol with 1.0x tryptic soy broth without dextrose (TSB) base nutrient. However, the medium for fermentation at optimum process variables contained 50 g/l glycerol. Into 125 ml serum bottles was dispensed 47.5 ml of the fermentation broth, and the bottles capped with sealed butyl rubber stopper. The headspaces were purged with nitrogen gas for about 2 min to create anaerobiosis, and the medium-containing bottles autoclaved at 121°C for 18 min. They were inoculated with the seed culture by using hypodermic syringe. The syringe needle was not removed but had a 0.45 μm gas-outlet filter fitted to its base. This prevented air from entering, but allowed gaseous products to escape, preventing pressure build-up that could interfere with the bacterial growth and function. The bottles were incubated in a rotary shaker at the desired cultural conditions described in the design of experiments. Samples were collected and prepared for HPLC analysis by filtering them through a 0.2 μm membrane filters.

### Effect of oxygen

The effect of oxygen was investigated using 25 ml bottles containing 10 ml of fermentation broth, which were incubated at 120 rpm and 37°C. Three different experimental set-ups were employed namely, anaerobic set (AN), aerobic set (AE), and one day anaerobic set (1dAN). The first set of fermentation vessels, the AN group, were purged with nitrogen after autoclaving, inoculated and then incubated for 72 h, having the filter-fitted needle on for gas outlet. Another set of vessels, the AE group, were not purged of air and the caps had the filter-fitted needles on, but the filter was a 0.45 μm gas inlet–outlet membrane (i.e., it permitted both gas inlet and outlet). This enabled air to enter the system as well as let the gaseous fermentation products to escape. These vessels were also inoculated and incubated for 72 h.

A third set of vessels, the 1dAN group, were treated like the first, inoculated and incubated like the rest. But after 24 h, the gas outlet filter was replaced with an inlet–outlet filter. Sample from each vessel was collected and analyzed with HPLC. For all fermentations in this work, the inoculum constituted 5% of the fermentation broth.

## Results

### Screening of important process variables by Taguchi design method

The influence of temperature, agitation speed, inoculum volume and pH on the production of ethanol was screened after 24 h and 120 h using Taguchi experimental design method. The result showed that after 24 h, agitation speed had the highest range of SN followed by inoculum volume within the tested range. However, after 120 h, temperature ranked first followed by agitation speed. This showed that time is also a factor that influenced the fermentation. Taking the average of 24 h and 120 h SN results, agitation speed had highest, followed by temperature. Initial pH was consecutively the least in the rank, signifying probably that the range of values tested was very close to the optimum pH value. Therefore temperature, agitation speed and time were used for the optimization step using full factorial design.

### Screening and optimization of process variables by the full factorial design

Full factorial design method was employed to further investigate the influence of temperature, time, and agitation speed (Table 2), as well as to determine the optimum values. Multiple regression analysis was applied to the experimental data to model the response variable, Y_EP_. Therefore, equation 1 was fitted to correlate the relationship between the variables and the response. It was restated to represent our independent variables as:

(2) $$\begin{array}{l} Y_{\mathrm{EP}}=\beta_{0+}\beta_{1}T+\beta_{2}T^{2}+\beta_{3}A+\beta_{4}A^{2}+\beta_{5}M\\ \;+\beta_{6}M^{2}+\beta_{7}\mathrm{AT} \end{array}$$

Excluding non-significant terms (p > 0.05) and plugging the values of the coefficients, the equation becomes:

(3) $$\begin{array}{l} Y_{\mathrm{EP}}=-25.0093+0.35113M-0.00{232M}^{2}\\ \;+4.43233T-0.0{58422T}^{2}-0.72278A\\ \;+0.00{229A}^{2} \end{array}$$

where M, T, and A are actual values of time (h), temperature (°C) and agitation speed (rpm), respectively, while Y_EP_ is the corresponding ethanol production (g/l).

From the analysis of variance (ANOVA), it was observed that the fitting model was very significant (*p* = 9.59x10^-11^). It was also found that the linear effect of time (*p* = 6.12x10^-5^), quadratic effect of time (*p* = 0.004), linear effect of temperature (*p* = 0.001), and quadratic effect of temperature (*p* = 0.002) on ethanol production from glycerol were very significant. Similarly, the linear effect of agitation speed (*p* = 2.46x10^-6^) as well as the quadratic effect of agitation speed (*p* = 2.91x10^-8^) were highly significant. However, the interaction of temperature and agitation (*p* = 0.101) had no significant effects on the ethanol production. The value of R^2^, the coefficient of determination, was 0.9463. This means that 94.63% of the variability in the response (dependent) variable, Y_EP_, is explained. The value of the R^2^_adj_, which tells whether adding a new coefficient would or would not improve the regression model, was also high (0.9266).

Equation 3 shows that the linear coefficient of temperature had positive value whereas its quadratic coefficient was negative. This implies that increase in temperature, other things being equal, would increase ethanol production at a steadily decreasing rate. On the other hand, the linear coefficient of agitation had a negative value while the quadratic coefficient was positive. The implication is that increasing agitation speed, while keeping other factors constant, would decrease ethanol production at a steadily increasing rate. Moreover, all the coefficients but β_1_ have values less than one. The fact that β_1_ > 4 indicates that the linear effect of temperature is dominant. Using the information in Eq.3, we found that the optimum temperature and agitation speed were 38°C and 200 rpm, respectively. The equation also indicated that the best incubation time was 72 h. These values were, therefore, used for fermentation of higher glycerol concentration (50 g/l). Meyer et al. ( 2011) reported that glycerol conversion and ethanol production by *E. aerogenes* were higher at 37°C than at room/ambient temperature (30°C) when initial glycerol concentration was higher than 15 g/l. We also deduced that running the fermentation beyond 48 h would only slightly increase ethanol production, while incubating beyond 72 h would not increase ethanol production any further.

### Fermentation of glycerol at optimum temperature and agitation

**Figure 1:**
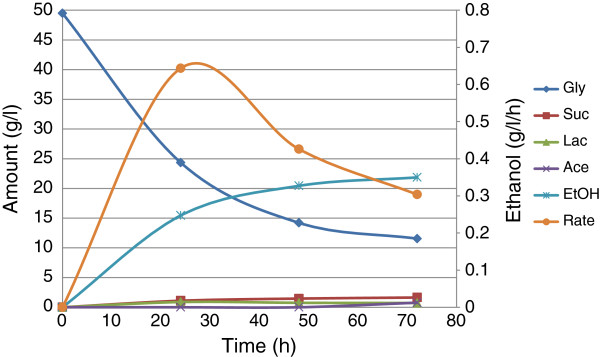
**Fermentation of pure glycerol by *E. aerogenes *S012 at optimum temperature and agitation speed.**

The result of fermentation at optimum temperature and agitation is presented in Figure 1. It shows that 20.4 g/l ethanol was produced from 50 g/l glycerol after 48 h, a yield of 1.16 mol/mol-glycerol and productivity (rate) of 0.43 g/l/h, while an additional 1.5 g/l (total of 21.9 g/l) was produced after 72 h, with a yield and productivity of 1.15 mol/mol-glycerol and 0.3 g/l/h, respectively.

This confirms the deductions made from equation 3 that extending the incubation time beyond 48 h would not make much difference in product formation. The fact that fermentation virtually stopped after 48 h suggests that an important factor is depleted in the system. It could be an accumulation of NADH, which is preventable by an electron acceptor such as oxygen or 1,3-Pdo. It could also be one or more of pH lowered below the tolerable limit for the organism, product inhibition, and depletion of nutrients. The first assumption—effect of oxygen—was, therefore, investigated.

### Effect of oxygen

**Figure 2:**
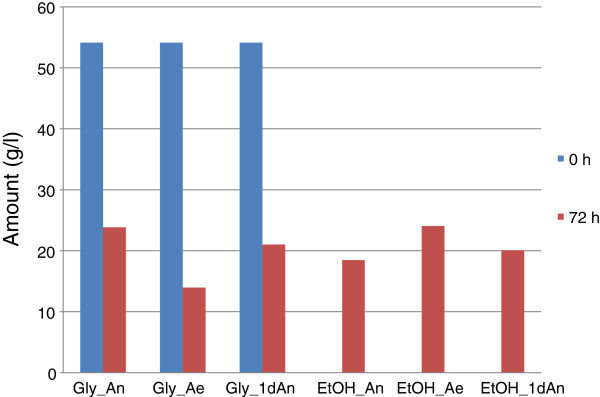
**Effect of oxygen on production of ethanol from glycerol by *E. aerogenes *S012.**

The effect of oxygen is summarized by Figure 2. The result showed that 24 g/l ethanol was produced from 50 g/l glycerol in the aerated fermentation, with productivity of 0.33 g/l/h after 72 h; 18.5 g/l was produced in the anaerobic process with productivity of 0.26 g/l/h; and 20.1 g/l produced in the anaerobic-aerobic process, a productivity of 0.28 g/l/h. This proved that some oxygen is required for optimum production of ethanol from glycerol by *E. aerogenes* S012. As stated before, small amounts (less than 2 g/l) of succinate, acetate and lactate were produced, while 1,3-Pdo was not produced.

**Figure 3:**
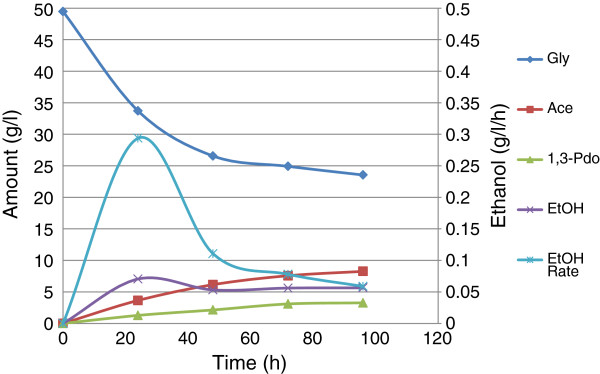
**Effect of excess air on the fermentation of glycerol by *E. aerogenes *S012 Optimized fermentation.**

However, when the reactor caps were loosely screwed to allow excess air into the fermentation vessels, acetate (7.6 g/l), ethanol (5.6 g/l), and 1,3-Pdo (3.1 g/l) were the only liquid products after 72 h (Figure 3). It could be seen from Figure 3 that the products were almost all formed in 48 h; after that very little was produced at 72 h, beyond which no further fermentation occurred. Glycerol utilization followed similar pattern, as the graph shows: only 25 g/l was utilized in 72 h.

**Figure 4:**
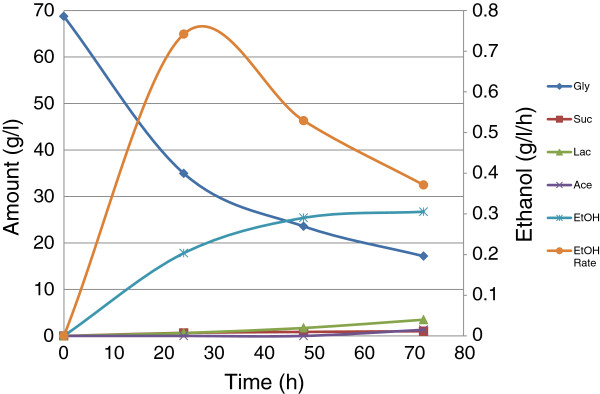
Fermentation of glycerol at aerated optimum conditions.

Fermentation at optimum temperature (38°C) and agitation speed (200 rpm) was repeated without purging the fermentation vessels with nitrogen gas. The fermentation medium contained 70 g/l glycerol and the needle fitted on the cap had gas inlet–outlet filter attached on its base. The result (see Figure 4) shows that over 45 g/l glycerol was utilized within 48 h, giving ethanol production, yield, and productivity of 25 g/l, 1.12 mol/mol-glycerol, and 0.53 g/l/h, respectively. The maximum productivity of 0.74 g/l/h was obtained after 24 h incubation. After 72 h, the glycerol utilization, ethanol production, ethanol productivity, and ethanol yield were 51.6 g/l, 26.2 g/l, 1.04 mol/mol-glycerol, and 0.37 g/l/h, respectively. This result proves that small amount of oxygen is required by the mutant *E. aerogenes* S012 for optimum conversion of glycerol to ethanol. The result also demonstrates that the organism’s activity is still greatly repressed after 48 h, confirming the deduction from Eq. 3.

## Discussion

The screening of important cultural factors by Taguchi method shows that initial pH had the least effect on the production of ethanol from glycerol by *E. aerogenes* S012. The reason could be that the range of pH values tested was very close to the optimum value. Ito *et al*. ( 2005) found pH 6.8 to be optimum for hydrogen and ethanol production by *E. aerogenes* HU-101, while Nakashimada *et al*. ( 2002) found pH 7.0 and 6.3 best for growth and hydrogen production, respectively, by *E. aerogenes* AY-2 strain. Therefore, best pH for fermentation by *E. aerogenes* S012 is between 6.3 and 6.8.

The best cultural conditions were 38°C, 200 rpm and 72 h, as deduced from Eq.3. However, Figure 1 shows that the fermentation virtually ceased after 48 h. This confirms another deduction from Eq.3 which found that running the fermentation beyond 48 h may not increase product formation significantly. However, we believe that this trend could also be an indication that the presence or depletion of some factor(s) adversely interfered with the activity of the biocatalyst. Among the factors suspected were lowered medium pH below the organism’s tolerable limit by metabolites, accumulation of NADH, product (ethanol) inhibition, and depletion of nutrients. It is important to keep the range of tested cultural conditions within the tolerance limit of the organism. For instance, temperature should be between 20-45°C and agitation between 120–200 rpm. Too high agitation speed might rupture the cell wall of the bacteria leading to death. Also temperatures outside the stated range may not support the growth of the organism. It is important to note that the response values and deductions of Eq.3 were based on experiments with feedstock concentration of 30 g/l, and may be considered qualitative values.

It could be seen that trace amounts of succinate, lactate, and acetate were produced as byproducts of the fermentation. These probably lowered the pH of the medium to a value at which the organism could not function anymore. For each set of fermentation, the pH was measured after every 24 h. The pH was about 5.9 after 24 h; then about 5.5 after 48 h, and the value stayed almost unchanged after 72 h. Meanwhile the initial pH was 7.0 ± 0.2.

Most enteric bacteria possess type 1 NAD^+^-dependent glycerol dehydrogenase (GDH-1), and ferment glycerol by oxidative and reductive pathways. In the oxidative pathway, glycerol is converted to dihydroxyacetone phosphate (DHAP) by two successive enzymes, namely GDH-1 and DHA kinase (DHAK), and NAD^+^ is reduced to NADH. DHAP then enters glycolysis to produce pyruvate, which can be broken down to various products depending on the organism.

In the reductive pathway, however, glycerol is dehydrated into 3-hydroxypropionaldehyde, 3-HPA, by glycerol dehydratase (GD). Then a NADH-linked 1,3-Pdo dehydrogenase (1,3-PdoDH) reduces 3-HPA to 1,3-Pdo and NADH is reoxidized to NAD^+^. Biomass yield during glycerol dissimilation also requires NAD^+^ for oxidation reactions, giving rise to NADH. In other words, microbial cells can only grow in a redox-balanced process in which NAD^+^ is regenerated from NADH. Although E*. coli* does not produce 1,3-Pdo like other *Enterobacteriaceae* do because it possesses type 2 GDH (GDH-2), it produces 1,2-pdo, which also does the work of regenerating NAD^+^ (Gonzalez et al. 2008). It follows that during biosynthesis of ethanol from glycerol by cells that don’t produce 1,2- or 1,3-pdo, cell growth releases a surplus of NADH, which inhibits further growth and cell function if not reoxidized. Thus for cell growth or glycerol metabolism, NAD^+^ concentration needs to be higher than NADH.

It follows that the key function of 1,3-pdo is to regenerate NAD^+^ reduced in the oxidative pathway or during cell growth. Therefore, species which do not synthesize 1,3-pdo need an alternative source of electron sink to regenerate NAD^+^. This could be achieved either by utilizing oxygen or by using complex compounds in the medium in which case they don’t synthesize biomass, thereby avoiding the necessity of NAD^+^ regeneration (Choi et al. 2011). Gonzalez et al. ( 2008) corroborated this when they reported that metabolism of glycerol in microbial species unable to synthesize 1,3-pdo takes place through a respiratory pathway that requires an external electron acceptor.

Figure 1 shows that *E. aerogenes* S012 produced neither 1,2-Pdo nor 1,3-Pdo at the experimental conditions, but gave small amounts of succinic, lactic, and acetic acids as the only organic coproducts. The screening experiments gave similar results (data not shown). Therefore, we assumed that there was an accumulation of NADH in the cells, which halted the assimilation of glycerol. Hence, there was need for an electron acceptor such as oxygen for regeneration of NAD^+^. Choi et al. ( 2011) discovered that *Kluyvera cryocrescens* S26 produced neither 1,2- nor 1,3-Pdo. This corroborates our report that it is possible for bacterial cells to produce ethanol from glycerol without co-producing propanediol (Pdo). *K. cryocrescens* S26 also coproduced H_2_ and small amounts of formic, lactic and succinic acids as the only organic acids.

The result shown in Figure 2 confirmed that low amount of oxygen was required by *E. aerogenes* S012 for optimum yield of ethanol from glycerol. This was also supported by Choi *et al*. ( 2011), Oh et al. ( 2011), and Jitrwung and Yargeau ( 2011). In their optimization research, Jitrwung and Yargeau ( 2011) found that *E. aerogenes* ATCC 35029 required a low oxygen concentration, rather than oxygen free conditions, to optimally produce hydrogen and ethanol from glycerol. They reported that *E. aerogenes* requires and consumes externally-supplied gaseous oxygen before using oxygen from decomposable organic or inorganic compounds/salts in the medium.

Choi et al. ( 2011) found that *K. cryocrescens* S26 was at its best in microaerobic condition. The bacterium produced 27 g/l ethanol with a productivity of 0.61 g/l/h at a low oxygen level. They reported that limited oxygen served as electron acceptor that consumed excess reducing equivalents (NADH) generated during biomass synthesis. However they noted that high oxygen supply would result in most of the carbon being integrated into cellular mass and converted to CO_2_, leading to poor metabolite formation. Choi et al. ( 2011) also confirmed that high oxygen supply decreased ethanol production by *K. cryocrescens* S26, but increased acetic and lactic acid products as well as encouraged excessive cell growth. They concluded that oxygen plays a key role in a switch between biomass formation and ethanol production pathways. Excess oxygen favors the former while low oxygen the later. Oh et al. ( 2011) arrived at similar conclusion: high aeration rate increased biomass accumulation but lowered ethanol production by *Klebsiella pneumoniae* GEM 167, while low aeration rate of 0.5 vvm (vessel volume/minute) gave highest ethanol production of 21.5 g/l and productivity of 0.93 g/l/h at 200 rpm and 37°C. In contrast to all these, however, Chen et al. ( 2003) reported that microaerobic condition (air at 0.4vvm) favored more cell growth and less ethanol production by *Klebsiella pneumoniae* DSM 2026 rather than anaerobic condition (nitrogen at 0.4 vvm). They noted that complete absence of air was more desirable for ethanol production by *K. pneumoniae* DSM 2026.

From the foregoing, presence of oxygen can enhance or disrupt ethanol production by many glycerol-metabolizing facultative anaerobes depending on the amount of air supplied. High oxygen supply enhances the generation of ATP by reducing NADH. Then ATP is used for biomass synthesis. Thus high oxygen level drives more carbon flux towards biomass production. But in microaerobic conditions, the limited oxygen converts NADH produced during cell growth into NAD^+^ while maintaining carbon flux into ethanol production. Obviously, *K. cryocrescens* S26 (Choi et al. 2011), *K. pneumoniae* GEM 167 (Oh et al. 2011), *E. aerogenes* ATCC 35029 (Jitrwung & Yargeau 2011), and *E. aerogenes* S012 (current work) follow the microaerobic pathway.

Ethanol is generally known to inhibit the growth of microorganisms. Therefore, ethanol exerts a product inhibition on the microbial agents of its biosynthesis. Although *Kluyvera cryocrescens* S26 produced high amounts of ethanol from glycerol, Choi et al. ( 2011) reported that 50 g/l ethanol in the medium reduced the growth of the bacterium by 80%, supporting the proposition. But our finding that *E. aerogenes* S012 survived (but did not grow) in a growth medium containing over 60 g/l ethanol suggests that ethanol product inhibition may not be the repressing factor to further metabolism of glycerol beyond 48 h.

The result shown in Figure 3 attested to the fact that excess air represses ethanol production. Just as Choi et al. ( 2011) reported higher levels of acetic and lactic acids, this report also found predominance of acetic, lactic and succinic acids in high supply of oxygen. Similarly, Hong et al. ( 2009) found that *E. coli* AC-521 produced 85.8 g/l lactic acid in 88 h under aerated fermentation.

The optimized fermentation (Figure 4) showed that best ethanol yield of 1.12 mol/mol-glycerol was produced after 48 h, although maximum productivity (0.74 g/l/h) was recorded after 24 h. We reported in a previous study (Nwachukwu et al. 2012) that the wild strain, *E. aerogenes* ATCC 13048, seemed to be at its best for utilizing glycerol within 48 h. The result in the current study showed that the mutant strain, *E. aerogenes* S012, retained this high ethanol yield (or glycerol-to-ethanol conversion efficiency) of the wild strain.

The better ethanol production observed in Figure 4 than the production observed in Figure 1 showed that low oxygen was required for optimum ethanol production by *E. aerogenes* S012. Ethanol yield of 1.12 mol/mol-glycerol was in excess of the theoretical maximum (1.0 mol/mol-glycerol). The reasons for this have been stated previously to be probably due to additional carbon source provided to the bacterium by the amino acids of the complex medium, or some unknown carbon/electron sources in the medium (Nwachukwu et al. 2012). Dharmadi et al. ( 2006) and Murarka et al. ( 2008) reported that *E. coli* needed to be supplemented with rich nutrients as tryptone or yeast extract in order to dissimilate glycerol. Yeast extract contains nitrogen and carbohydrates. Therefore, as Choi et al. ( 2011) also noted, those carbohydrates may be utilized by the microbial cells as carbon source for ethanol production and biomass yield.

Several researchers have reported using recombinant species to ferment glycerol. Yang et al. ( 2007) found that engineered *Klebsiella oxitoca* M5al produced 19.5 g/l ethanol at the rate of 0.56 g/l/h; Durnin *et al*. ( 2009) reported a production of 20.7 g/l with a productivity of 0.22 g/l/h by a recombinant *E. coli*; and Oh et al. ( 2011) used a genetically modified *K. pneumoniae* to produce 25 g/l ethanol from glycerol. Cheng et al. ( 2007) reported that a wild *K. pneumoniae* M5al produced 18 g/l ethanol from pure glycerol, with a productivity of 0.28 g/l/h in a N_2_ gas-created anoxia. This work found that non engineered mutant, *E. aerogenes* S012, produced 25.4 g/l ethanol from pure glycerol in 48 h, with productivity of 0.53 g/l/h and yield of 1.12 mol/mol-glycerol. This means that 56.23% of the used glycerol was converted to ethanol.

It is well known that manipulation of culture parameters is a process-based improvement strategy to optimize product formation (Yazdani and Gonzalez, 2007), while detection of mutants, genetic operations, and metabolic pathway control are strain-based improvement strategies. The use of process-based protocols to optimize product formation is preferable to the use of recombinant DNA mechanisms. The reason is that commercial/industrial use of genetically modified (GM) organisms is restricted by government regulations through EPA and USDA (D. Glass Associates Inc 2012). D. Glass Associates Inc ( 2012)) reported that biofuels companies may experience significant interference and restrictions due to the government regulations on the testing and commercial use of GM microorganisms, algae, or transgenic plants being developed for biofuels applications. Although the GM products could be more efficient for commercial applications, Bell and Attfield ( 2009) noted that these regulatory constraints can increase the capital and running costs of biofuels production. Therefore, improving biological catalysts of bioethanol production by measures that do not include genetic engineering is very desirable.

This work used TSB for all fermentations. According to Gullapalli et al. ( 2007), the type of medium used in fermentation greatly influences the type and quantity of products formed. Choi et al. ( 2011) found that yeast extract was best for biomass accumulation and ethanol yield by *K. cryocrescens* S26. However, Gullapalli et al. ( 2007), in their bioproduction of D-psicose by *E. aerogenes*, discovered that the highest transformation rate was obtained when tryptic soy broth (TSB) was the fermentation broth. In a preliminary study, Banna and Ouro (2008, unpublished) used Luria Bertani (LB) broth supplemented with tryptone and yeast extract as fermentation medium for *E. aerogenes*. They found that less than 10 g/l glycerol was utilized after 48 h, producing about 5 g/l ethanol. Although this represented a yield equivalent to theoretical maximum of 1 mol/mol-glycerol, the ethanol production, ethanol productivity, and glycerol utilization were very poor. The poor fermentation was probably due to the type of medium used or excess air in the system.

In conclusion, *Enterobacter aerogenes* S012 promises to be good and efficient biocatalyst for conversion of glycerol to ethanol. It produced 25.4 g/l ethanol within 48 h at optimum temperature and agitation speed of 38°C and 200 rpm, respectively, under a low amount of oxygen. It also demonstrated an ability to produce fewer co-products at trace concentrations and complete absence of 1,3-Pdo. This will make ethanol extraction/purification easier and more economically efficient. Previously, the wild strain, *E. aerogenes* ATCC 13048, was reported to be effective at converting low concentrations of recovered glycerol into ethanol (Nwachukwu et al. 2012). Therefore, we believe that the mutant strain, *E. aerogenes* S012, will be even more effective in converting higher concentrations of recovered glycerol into ethanol. Further work is necessary to verify this assumption. Moreover, the ability of this *E. aerogenes* S012 to convert glycerol to ethanol at effectiveness of 1 mol ethanol/mol glycerol in high glycerol-containing medium is potentially of great importance to biofuels industry. This ability makes the invention a potentially excellent biocatalyst for industrial conversion of glycerol to ethanol. Since the technology involves using biocatalyst developed by adaptive mutation, it is not subject to government regulations of GM products.

It was also observed in this work that the pH of the fermentation medium dropped to a value below 5.6 from the initial value of 7.0 ± 0.2 after 48 h. Additional work is necessary to confirm that *E. aerogenes* S012 is at its best for ethanol production at pH 6.3-6.8. Finally, it has been reported that hydrogen production is associated with ethanol production during microbial glycerol metabolism (Varrone et al. 2012). Choi et al. ( 2011) and Ito et al. ( 2005) also corroborated this during the fermentation of crude glycerol by the bacteria *K. cryocrescens* S26 and *E. aerogenes* HU-101, respectively. Therefore, further research is necessary to determine the amount of hydrogen produced in the glycerol metabolism by *E. aerogenes* S012.

## Competing interest

The authors declare that they have no conflict of interests.

## References

[B1] Bell PJL Attfield PV Breakthrough in yeast for making bio-ethanol from lignocellulosics 2009 http://www.eri.ucr.edu/ISAFXVCD/ISAFXVAF/YMBL.pdf

[B2] Chapman JA Correll RL Ladd JN Removal of soluble organic carbon from winery and distillery wastewaters by application to soil Aust J Grape Wine Res 1995 1 39 47

[B3] Chen X Zhang DJ Qi WT Gao SJ Xiu ZL Xu P Microbial fed-batch production of 1,3-propanediol by Klebsiella pneumoniae under micro-aerobic conditions Appl Microbiol Biotechnol 2003 63 143 6 12908084 10.1007/s00253-003-1369-5

[B4] Cheng KK Zhang JA Liu DH Sun Y Liu HJ Yang MD Xu JM Pilot-scale production of 1,3-propanediol using Klebsiella pneumoniae Process Biochem 2007 42 4 740 744

[B5] Choi WJ Glycerol-based biorefinery for fuels and chemicals Recent Pat Biotechnol 2008 2 173 180 19075864 10.2174/187220808786241006

[B6] Choi WJ Hartono MR Chan WH Yeo SS Ethanol production from biodiesel-derived crude glycerol by newly isolated Kluyvera cryocrescens Appl Microbiol Biotechnol 2011 89 1255 1264 21212944 10.1007/s00253-010-3076-3

[B7] D. Glass Associates Inc Government and regulatory affairs—Use of modified microorganisms, algae or transgenic plants for biofuels 2012 http://www.dglassassociates.com/REGUL/biofuels.htm

[B8] Dharmadi Y Murarka A Gonzalez R Anaerobic fermentation of glycerol by Escherichia coli: a new platform for metabolic engineering Biotechnol Bioeng 2006 94 5 821 829 16715533 10.1002/bit.21025

[B9] Durnin G Clomburg J Yeates Z Alvarez PJJ Zygourakis K Campbell P Gonzalez R Understanding and harnessing the microaerobic metabolism of glycerol in Escherichia coli Biotechnol Bioeng 2009 103 148 161 19189409 10.1002/bit.22246

[B10] Fan X Burton R Zhou Y Glycerol (Byproduct of Biodiesel Production) as a Source for Fuels and Chemicals—Mini Review Open Fuels Energy Sci J 2010 3 17 22

[B11] Fraley S Oom M Terrien B Zalewski J Design of experiments via taguchi methods: orthogonal arrays 2007 Retrieved November 4, 2009, from http://controls.engin.umich.edu/wiki/index.php/Design_of_experiments_via_taguchi_methods:_orthogonal_arrays

[B12] Ghosh D Sobro IF Hallenbeck PC Stoichiometric conversion of biodiesel derived crude glycerol to hydrogen: Response surface methodology study of the effects of light intensity and crude glycerol and glutamate concentration Bioresour Technol 2012 106 154 160 22206915 10.1016/j.biortech.2011.12.021

[B13] Gonzalez R Murarka A Dharmadi Y Yazdani SS A new model for the anaerobic fermentation of glycerol in enteric bacteria: trunk and auxiliary pathways in Escherichia coli Metab Eng 2008 10 234 245 18632294 10.1016/j.ymben.2008.05.001

[B14] Gullapalli P Takata G Poonperm W Rao D Morimoto K Akimitsu K Tajima S Izumori K Bioproduction of D-psicose from allitol with Enterobacter aerogenes IK7: a new frontier in rare ketose production Biosci Biotechnol Biochem 2007 71 12 3048 3054 18071247 10.1271/bbb.70450

[B15] Hazimah AH Ooi TL Salmiah A Recovery of glycerol and diglycerol from glycerol pitch J Oil Palm Res 2003 15 1 1 5

[B16] Hong AA Cheng KK Peng F Zhou S Sun Y Liu CM Liu DH Strain isolation and optimization of process parameters for bioconversion of glycerol to lactic acid J Chem Technol Biotechnol 2009 84 1576 1581

[B17] Ito T Nakashimada Y Senba K Matsui T Nishio N Hydrogen and ethanol production from glycerol-containing wastes discharged after Biodiesel manufacturing process J Biosci Bioeng 2005 100 3 260 265 16243274 10.1263/jbb.100.260

[B18] Jitrwung R Yargeau V Optimization of media composition for the production of biohydrogen from waste glycerol Int J Hydrog Energy 2011 36 9602 9611

[B19] McCoy M Glycerin surplus Chem Eng News 2006 84 6 7 8

[B20] Meyer P Pankaew S Boonsawang P Tongurai C Anaerobic fermentation of crude glycerol to produce value-added products Appl Eng Agric 2011 27 4 655 662

[B21] Murarka A Dharmadi Y Yazdani SS Gonzalez R Fermentative utilization of glycerol in Escherichia coli and its implications for the production of fuels and chemicals Appl Environ Microbiol 2008 74 1124 1135 18156341 10.1128/AEM.02192-07 PMC2258577

[B22] Nakashimada Y Rachman MA Kakizono T Nishio N Hydrogen production of Enterobacter aerogenes altered by extracellular and intracellular redox states Int J Hydrog Energy 2002 27 1399 1405

[B23] Nicol RW Marchand K Lubitz WD Bioconversion of crude glycerol by fungi Appl Microbiol Biotechnol 2012 93 1865 1875 22322872 10.1007/s00253-012-3921-7

[B24] Nwachukwu RES Shahbazi A Wang L Ibrahim S Worku M Schimmel K Bioconversion of glycerol to ethanol by a mutant Enterobacter aerogenes AMB Express 2012 2 20 10.1186/2191-0855-2-20 22455837 PMC3350409

[B25] OECD/FAO Biofuels. OECD-FAO Agricultural Outlook 2011–2020 2011 Retrieved April 27, 2012, from http://www.oecd.org/document/0/0,3746,en_36774715_36775671_47877696_1_1_1_1,00.html

[B26] Oh BR Seo JW Heo SY Hong YK Luo LH Joe MH Park DH Kim CH Efficient production of ethanol from crude glycerol by a Klebsiella pneumoniae mutant strain Bioresour Technol 2011 102 3918 3922 21186120 10.1016/j.biortech.2010.12.007

[B27] Pachauri N He B Value-added utilization of crude glycerol from biodiesel production: A survey of current research activities 2006 ASABE Annual Meeting Presentation, Portland, OR

[B28] Rossi DM da Costa JB de Souza EA Peralba MR Ayub MAZ Bioconversion of residual glycerol from biodiesel synthesis into 1,3-propanediol and ethanol by isolated bacteria from the environmental consortia Renew Energy 2012 39 223 227

[B29] Sarma SJ Brar SK Sydney EB Bihan YL Buelna G Soccol CR Microbial hydrogen production by bioconversion of crude glycerol: A review Int J Hydrog Energy 2012 37 6473 6490

[B30] Sims B US, Argentina surge in world biodieselproduction rankings. Biodiesel Magazine 2011 Retrieved September 04, 2012, from http://www.biodieselmagazine.com/articles/8254/us-argentina-surge-in-world-biodiesel-production-rankings

[B31] Selembo PA Perez JM Lloyd WA Logan BE High hydrogen production from glycerol or glucose by electrohydrogenesis using microbial electrolysis cells Int J Hydrog Energy 2009 34 5373 5381

[B32] Varrone C Giussani B Izzo G Massini G Marone A Signorini A Wang A Statistical optimization of biohydrogen and ethanol production from crude glycerol by microbial mixed culture Int J Hydrog Energy 2012 30 1 10

[B33] Yang G Tian J Li J Fermentation of 1, 3-propanediol by a lactate deficient mutant of Klebsiella oxytoca under microaerobic conditions Appl Microbiol Biotechnol 2007 73 1017 1024 16960737 10.1007/s00253-006-0563-7

[B34] Yazdani SS Gonzalez R Anaerobic fermentation of glycerol: a path to economic viability for the biofuels industry Curr Opin Biotechnol 2007 18 213 219 17532205 10.1016/j.copbio.2007.05.002

